# Inverse Regulation of Serum Osteoprotegerin and B-Type Natriuretic Peptide Concentrations by Free Fatty Acids Elevation in Young Healthy Humans

**DOI:** 10.3390/nu14040837

**Published:** 2022-02-17

**Authors:** Marta Dobrzycka, Adrian Kołakowski, Magdalena Stefanowicz, Natalia Matulewicz, Agnieszka Nikołajuk, Monika Karczewska-Kupczewska

**Affiliations:** 1 Department of Internal Medicine and Metabolic Diseases, Medical University of Białystok, 15-276 Białystok, Poland; marti.rydzewska@gmail.com (M.D.); adriankolakowski17@gmail.com (A.K.); 2 Department of Metabolic Diseases, Medical University of Białystok, 15-276 Białystok, Poland; magdalenazielinska.umb@gmail.com (M.S.); natalia.kaminska85@gmail.com (N.M.); 3 Department of Prophylaxis of Metabolic Diseases, Institute of Animal Reproduction and Food Research, Polish Academy of Sciences, 10-748 Olsztyn, Poland; agstepien@poczta.onet.pl

**Keywords:** osteoprotegerin, insulin resistance, B-type natriuretic peptide, metabolic syndrome, hyperinsulinemic-euglycemic clamp

## Abstract

Osteoprotegerin (OPG) and B-type natriuretic peptide (BNP) are cardiovascular risk factors, interrelated with each other, with possible associations with insulin sensitivity and glucose homeostasis. The aim of this study was to assess association between OPG and BNP concentrations in a young healthy population, their relation to insulin sensitivity and obesity and their regulation by hyperinsulinemia and serum free fatty acids (FFA) elevation. The study group consisted of 59 male volunteers, 30 of whom were of a normal weight (BMI < 25 kg/m^2^), and 29 were overweight/obese (BMI > 25 kg/m^2^). Insulin sensitivity was assessed with the 2-h hyperinsulinemic-euglycemic clamp (HEC). In the subgroup of 20 subjects, the clamp was prolonged to 6 h. After one week, another 6-h clamp, with concurrent Intralipid/heparin infusion, was performed. Serum OPG was positively associated with insulin sensitivity (*p* = 0.002) and negatively with BMI (*p* = 0.019) and serum BNP (*p* = 0.025). In response to 6-h hyperinsulinemia, circulating BNP decreased (*p* < 0.001). In response to HEC with Intralipid/heparin infusion, OPG decreased (*p* < 0.001) and BNP increased (*p* < 0.001). Our data show that OPG and BNP are differentially regulated by FFA, which suggests their association with lipid-induced insulin resistance. The assessment of these cardiovascular risk factors should take into account both long-term and short-term effects associated with insulin resistance.

## 1. Introduction

Insulin resistance (IR) is associated with an increased risk of type 2 diabetes and cardiovascular diseases [1]. Osteoprotegerin (OPG) and B-type natriuretic peptide, initially called brain natriuretic peptide (BNP), are nonclassical cardiovascular risk factors, interrelated with each other [2], with possible associations with insulin sensitivity and glucose homeostasis.

OPG is a soluble member of the tumor necrosis factor (TNF) receptor superfamily, which exerts pleiotropic effects on bone metabolism, vascular inflammation and endocrine function. In detail, OPG inhibits osteoclastogenesis and bone resorption by acting as a decoy receptor for Receptor Activator for Nuclear Factor κ B Ligand (RANKL) in the RANK/RANKL/OPG axis [3]. It is secreted by diverse tissues, including the lung, kidney, immune tissues and the cardiovascular system [4]. According to animal studies, OPG exerts a protective effect in vasculature by the prevention of both plaque burden progression and plaque calcification [3]. The clinical studies indicate that the serum OPG levels increase in association with vascular calcification, coronary artery disease [5] and heart failure [2]. Furthermore, recent data suggest that the elevated concentration of OPG can be regarded as a promising potent and independent predictor of cardiovascular disease in high-risk populations [6]. Recent research suggests the linkage between OPG and insulin sensitivity, as well as glucose homeostasis. The numerous studies have shown the elevated concentrations of OPG in diabetic individuals [4,7,8]. However, the relation between the blood concentration of OPG and the homoeostasis model assessment of insulin resistance (HOMA-IR) has significantly varied in recent studies [8,9,10]. There is no clear knowledge about the relationship between OPG and obesity, which is a risk factor for diabetes and cardiovascular diseases. In the literature, we can find works describing the lack of a correlation between OPG and obesity [11,12], as well as describing a positive correlation between these factors [13].

BNP and N-terminal pro B-type natriuretic peptide (NTproBNP) are neurohormones, which are believed to be interchangeable and may be used interchangeably for research purposes. The human BNP gene, located on chromosome 1, encodes the 108 amino acid proBNP prohormone. It then breaks down into the 32-amino acid hormone BNP and the N-terminal part of the prohormone, known as NT-proBNP. This causes the release of active BNP and the inactive part, which is NT-proBNP, into the blood [14]. BNP and NTproBNP are released in response to a cardiac muscle stretch, ventricular ischemia or volume overload. They mediate their effects through the renin–angiotensin–aldosterone system reflecting neuroendocrine activation. Intravenous infusion of active BNP, which we decided to study, causes a fall in plasma rennin activity and inhibits the plasma aldosterone response to angiotensin II [15]. Cardiac B-type natriuretic peptides are known as well-established biomarkers widely used for diagnosis and prognosis determination of heart failure in clinical medicine [16]. Even in the groups without heart failure, the higher BNP concentrations provide information about future cardiovascular risk [17]. However, there are many factors that affect BNP levels in individuals, including gender and age, renal disease and hypertension [18]. Moreover, the epidemiological studies have demonstrated lower plasma concentrations of natriuretic peptides in obese individuals than in normal-weight ones, despite the higher prevalence of hypertension, diabetes and left ventricular hypertrophy [19]. In the case of patients with diabetes, higher plasma levels of natriuretic peptides have been identified as the strongest independent predictor of cardiovascular mortality [20]. The paradox of both low and increased levels of natriuretic peptides in insulin-resistance-mediated diseases, such as obesity and type 2 diabetes, is still not fully understood.

The recent studies have shown a positive correlation between OPG and NT-proBNP, thus synergistically increasing heart failure risk [2]. Moreover, BNP and OPG may be biochemical markers of cardiovascular diseases such as aortic pathology and heart failure [21,22,23]. There are also studies showing the increased concentration of both substances in myocardial infarction [24]. The reason for this association is still unclear. Moreover, data referring to the relationship between metabolic syndrome and natriuretic peptides or OPG are controversial, according to studies showing both increased and reduced blood levels of OPG and BNP. However, human studies were carried out mostly in elderly people with severe metabolic abnormalities and other diseases (i.e., heart diseases, type 2 diabetes), and the different results obtained are mainly attributed to different study populations. Furthermore, there are scarce data based on dynamic methods measuring insulin sensitivity. It is relevant to conduct research on young and healthy subjects without overt metabolic disturbances to avoid the influence of possible confounding factors.

Therefore, the aim of this study was to assess the association between serum OPG and BNP concentrations in a young healthy population and their relation to insulin sensitivity and obesity. Furthermore, we decided to study the influence of hyperinsulinemia and serum free fatty acids (FFA) elevation, parameters strongly associated with insulin resistance, on circulating OPG and BNP in this population.

## 2. Materials and Methods

### 2.1. Study Participants

The study group consisted of 59 young, healthy male volunteers, 30 of whom were of a normal weight (body mass index, BMI < 25 kg/m^2^), and 29 were overweight/obese (BMI > 25 kg/m^2^). All study individuals were without severe disorders or extreme obesity. They were not taking any medications and were not smoking. Volunteers were excluded if they had any inflammatory disorders or had taken anti-inflammatory drugs within the last 3 months. Anthropometric measurements were performed as previously shown [25]. Participants underwent clinical examination and appropriate biochemical tests. The BMI of the individuals had remained stable for at least 3 months before the research. The study protocol was approved by the local ethics committee of the Medical University of Bialystok. Written informed consent was obtained from all volunteers before their participation in the study.

### 2.2. Glucose Tolerance and Insulin Sensitivity

In a standard oral glucose tolerance test (OGTT), all subjects had normal glucose tolerance according to World Health Organization criteria. Next, after one week, insulin sensitivity (M) was assessed with the 2-h hyperinsulinemic-euglycemic clamp (HEC) technique, as previously described [26], and was calculated per fat-free mass (ffm). In the subgroup of 20 male participants (9 were normal-weight, and 11 were overweight/obese), the clamp was prolonged to 6 h. After one week, another 6-h clamp, with concurrent Intralipid/heparin infusion, was performed as described [25]. No difference in the steady-state insulin concentration between these protocols was seen.

### 2.3. Biochemical Procedures

Plasma glucose, serum lipids and insulin were measured as previously described [26,27]. Serum OPG was measured with an enzyme immunoassay kit (Biomedica Medizinprodukte, GmbH& Co KG, Vienna, Austria) with the detection limit of 0.07 pmol/L and with intra-assay and inter-assay coefficients of variation (CVs) below 3 and 5%, respectively. Serum BNP was measured with an enzyme-linked immunosorbent assay (ELISA) Kit (Phoenix Pharmaceuticals Inc., Burlingame, CA, USA) with the detection limit of 0.42 ng/mL and with intra-assay and inter assay CVs below 10 and 15%, respectively.

### 2.4. Statistical Analysis

Statistics were performed with the STATISTICA 12.5 program (StatSoft, Krakow, Poland). All data are presented as the mean ± standard deviation. Variables which did not have a normal distribution were log transformed before analyses. For data presentation, these variables were antilog transformed again to absolute values. The differences between normal-weight and overweight/obese individuals were analyzed with unpaired Student’s t-tests. Differences in the serum OPG or BNP concentrations during both clamps without and with Intralipid/heparin infusions were assessed with a repeated-measures ANOVA with a post-hoc Tukey test. Time × group interactions were also studied with a repeated-measures ANOVA, where a nonsignificant interaction indicated that no difference in the estimated effect was observed between the groups. The relationships between variables were studied with the Pearson product moment correlation analysis and with a multiple regression analysis. The level of significance was accepted as *p* < 0.05.

## 3. Results

**Figure 1:**
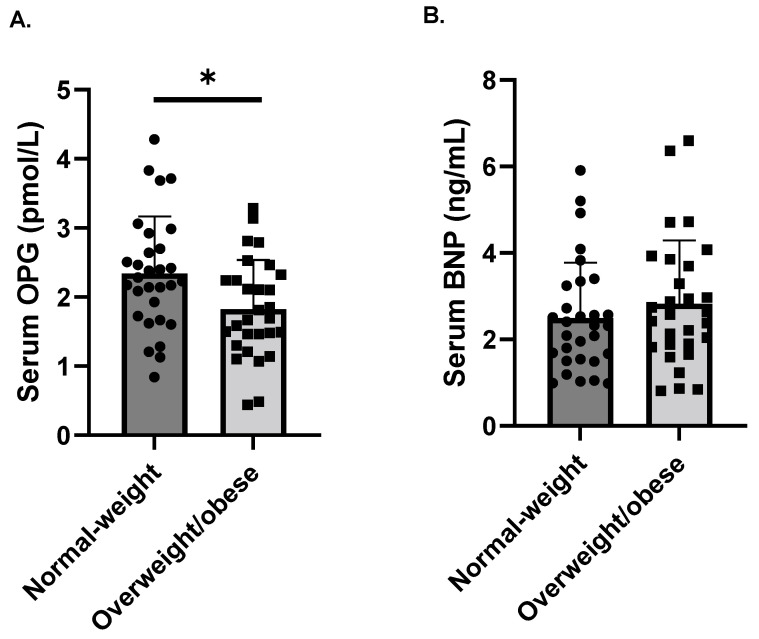
Circulating OPG (**A**) and BNP (**B**) concentrations, according to BMI (normal-weight: BMI < 25 kg/m^2^ and overweight/obese: BMI ≥ 25 kg/m^2^). Values are given as means ± SD, * *p* < 0.05.

The baseline characteristics of the study group were divided into normal-weight and overweight/obese subgroups, and the results of the laboratory tests are presented in Table 1. Insulin sensitivity was decreased in the overweight/obese group in comparison with the normal-weight group (*p* = 0.012). Importantly, the fasting OPG levels were significantly lower in overweight/obese subjects compared to normal-weight subjects (*p* = 0.013, Figure 1A). We did not observe a difference in the serum BNP concentration between subgroups (Figure 1B).

### 3.1. The Relationship of Circulating OPG and BNP with Other Estimated Parameters

Serum OPG was positively associated with insulin sensitivity (r = 0.40, *p* = 0.002) and negatively with BMI (r = −0.30, *p* = 0.019), waist circumference (r = −0.26, *p* = 0.049), % body fat (r = −0.27, *p* = 0.036) and serum BNP concentration (r = −0.29, *p* = 0.025). The multiple regression analysis revealed that OPG was associated with insulin sensitivity and BNP independently of BMI (β = 0.30, *p* = 0.016 and β = 0.32, *p* = 0.02, respectively). Serum BNP was positively correlated with systolic blood pressure (r = 0.31, *p* = 0.018). This correlation was also independent of BMI (β = 0.30, *p* = 0.01).

### 3.2. The Effects of Hyperinsulinemia without or with Intralipid/Heparin Infusion on Circulating OPG Levels

**Figure 2:**
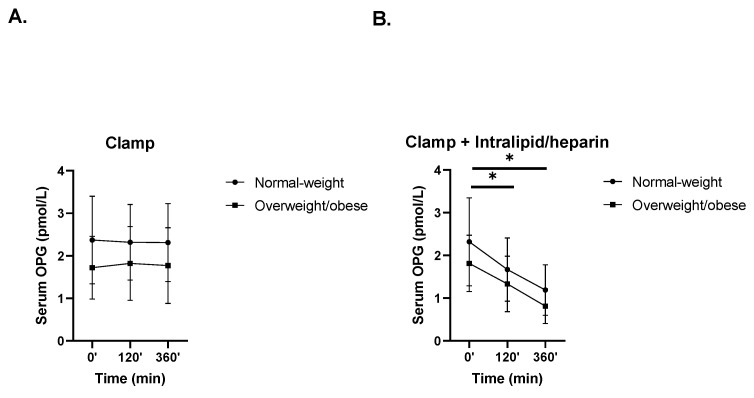
Effects of hyperinsulinemia and hyperinsulinemia with Intralipid/heparin infusion on circulating OPG concentrations in the normal-weight and overweight/obese groups [25]. Serum OPG levels in both subgroups during (**A**) HEC and (**B**) HEC with Intralipid/heparin infusion (vs. 0 min, * *p* < 0.05). Values are given as means ± SD.

In response to hyperinsulinemia during 6-h HEC, circulating OPG was stable throughout this study, both in the normal-weight group and in the overweight/obese group (Figure 2A). Conversely, during the 6-h HEC with Intralipid/heparin infusion, OPG significantly dropped similarly in the normal-weight group (from 2.32 ± 1.03 pmol/L to 1.67 ± 0.74 pmol/L at 120 min (*p* = 0.045), then to 1.19 ± 0.59 pmol/L at 360 min (*p* = 0.001)) and in the overweight/obese group (from 1.81 ± 0.66 pmol/L to 1.33 ± 0.65 pmol/L at 120 min (*p* = 0.006), then to 0.91 ± 0.41 pmol/L at 360 min (*p* = 0.0001); time × group interaction *p* = 0.93) (Figure 2B).

### 3.3. The Effects of Hyperinsulinemia without or with Intralipid/Heparin Infusion on Circulating BNP Levels

**Figure 3:**
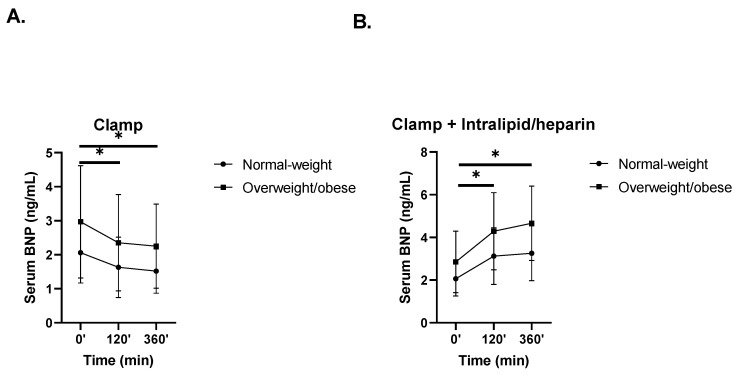
Effects of hyperinsulinemia and hyperinsulinemia with Intralipid/heparin infusion on circulating BNP concentrations in the normal-weight and overweight/obese groups [25]. Serum BNP levels in both subgroups during (**A**) HEC and (**B**) HEC with Intralipid/heparin infusion (vs. 0 min, * *p* < 0.05). Values are given as means ± SD.

In response to hyperinsulinemia during the 6-h HEC, circulating BNP significantly decreased both in the normal-weight group (from 2.06 ± 0.89 pg/mL to 1.63 ± 0.89 pg/mL at 120 min (*p* = 0.014), then to 1.52 ± 0.65 pg/mL at 360 min (*p* = 0.007)) and in the overweight/obese group (from 2.97 ± 1.65 pg/mL to 2.35 ± 1.42 pg/mL at 120 min (*p* = 0.031), then to 2.25 ± 1.24 pg/mL at 360 min (*p* = 0.007), time × group interaction *p* = 0.90) (Figure 3A). The opposite effect was observed during the 6-h HEC with Intralipid/heparin infusion, in which BNP significantly increased both in the normal-weight group (from 2.06 ± 0.81 pg/mL to 3.12 ± 1.33 pg/mL at 120 min (*p* = 0.02), then to 3.26 ± 1.29 pg/mL at 360 min (*p* = 0.007)) and in the overweight/obese group (from 2.85 ± 1.44 pg/mL to 4.29 ± 1.81 pg/mL at 120 min (*p* = 0.004), then to 4.66 ± 1.74 pg/mL at 360 min (*p* = 0.0004); time × group interaction *p* = 0.89) (Figure 3B).

## 4. Discussion

Based on our knowledge, our study is one of the first studies which investigates the relationship between OPG and BNP levels, cardiovascular risk factors and insulin sensitivity in healthy individuals. Many factors may have an influence on the secretion of OPG and BNP, such as the extent of obesity, drugs, glycemic status, other disorders and complications. In this study, individuals were young without morbid obesity and overt metabolic disturbances; thus, these may strengthen the analysis.

Furthermore, we demonstrated the influence of hyperinsulinemia with and without Intralipid/heparin infusion on serum OPG and BNP concentrations in this population. This is one of the first studies in which we investigated the effect of FFA on the concentrations of OPG and BNP in a population of healthy individuals. The main finding of our study was that hyperinsulinemia during 6-h HEC did not affect OPG levels and decreased BNP levels in young, both normal-weight and overweight/obese, apparently healthy subjects. Moreover, our study provides new evidence that during the 6-h HEC with Intralipid/heparin infusion, serum OPG concentrations significantly decreased, while BNP levels increased in both groups.

In our study, OPG was reduced in overweight or obese people and was negatively correlated with BMI, waist circumference and the amount of fat mass. Other authors showed a positive correlation between OPG and the above parameters in the group of patients with metabolic syndrome [13]. The values of OPG we observed in healthy people (2.32 ± 1.03 pmol/mL) were similar to those observed in control groups in other studies (1.95 pmol/mL ±  0.75 and 2.2 pmol/L, range: 1.4–6.0) [28,29]. Moreover, some researchers have even revealed that BMI does not correlate with OPG levels [12,30]. Kotanidou et al. showed that OPG levels did not differ significantly between the obese group and the control group. Obesity is often accompanied by other severe diseases modifying the presented mechanism, which may lead to a misconception of the real influence of obesity on the concentration of OPG. Thus, it can be concluded that the increases in OPG concentrations may be caused by the activation of compensatory mechanisms for existing diseases or by damage induced by an occurring disease, such as heart failure or diabetes type 2.

Similar to our data, reduced insulin sensitivity is associated with a decrease in OPG levels in healthy individuals but that are over 50 years-old. In the mentioned study, the presence of type 2 diabetes was an exclusion factor; however, the insulin sensitivity was measured by the quantitative insulin-sensitivity check index (QUICKI) [11]. The inverse correlation of the HOMA-IR and OPG concentration was found in 74 healthy young individuals without concomitant diseases, and OPG concentrations were decreased in obese subgroup [9]. In contrast, a positive correlation between increased insulin resistance and OPG levels was observed in patients with metabolic syndrome and established type 2 diabetes [31,32]. There are also studies in the literature describing the correlations of OPG with both BMI and insulin resistance [13]. Our data show that insulin resistance, and not BMI, is the main factor associated with circulating OPG, even when there are no overt metabolic disturbances. The coexistence of metabolic syndrome and insulin resistance significantly increases OPG levels, suggesting that a more severe metabolic state in a patient results in greater increases in OPG [33]. In our study, we used HEC, which is the golden standard in this type of experiment. It is noteworthy to mention that during HEC, the normal plasma glucose level is held; thus, we may eliminate the potential effect of hyperglycemia on circulating OPG levels. Due to the complexity of the process, there are only several studies on OPG using this technique, which significantly increases the credibility of our research. The method of the measurement of insulin sensitivity and the different inclusion criteria in the mentioned studies may be of significance.

The intravenous infusion of insulin during the 6-h HEC did not cause significant changes in the concentration of OPG. There is a study that describes that insulin reduces the concentration of OPG [34]. In contrast to our study, the individuals were over 50 years-old. It is well known that short-term Intralipid/heparin infusion essentially increases FFA concentrations and leads to insulin resistance by reducing peripheral glucose uptake and down-regulating intracellular insulin signaling [35]. To examine the impact of lipid-induced insulin resistance on circulating OPG in humans, we investigated the changes of the serum OPG level during lipid infusion combined with HEC. We observed that circulating OPG dropped significantly throughout this clamp. There are no studies presenting the results of any similar design of the methods. We can speculate that our results suggest that the decreased OPG concentrations are directly associated with lipid effects, rather than insulin. The data on the positive correlation between OPG and insulin sensitivity correspond well with the findings that FFA elevation decreases serum OPG, together with inducing insulin resistance.

In our study, we did not notice significant changes in BNP in the studied groups; there are numerous studies in the literature describing the relationship between BNP and obesity, but the results are inconsistent. The presence of obesity, type 2 diabetes or metabolic syndrome leads to a significant reduction in BNP concentrations and the development of natriuretic impairment [36,37,38]. Wang et al. reported that the negative effects of type 2 diabetes and obesity on the concentration of natriuretic peptides were additive [39]. The above works relate to patients with metabolic diseases, while Costello-Boerrigter et al. showed, in a multivariate analysis, that BMI had no significant effect on NT-proBNP levels in a group of 746 healthy people [40]. It follows that obesity itself does not have to affect the levels of BNP and NTproBNP, but it only predisposes one to the factors causing these changes in the concentration. We have also shown that BNP levels are positively correlated with systolic blood pressure, independently of BMI. The obtained results are consistent with the studies by Kohno et al. conducted on patients with left ventricular hypertrophy and hypertension [41]. The described positive corrections of BNP and blood pressure usually concern patients with diagnosed hypertension, while our results refer to the group of healthy patients without hypertension.

Furthermore, we also found that acute hyperinsulinemia decreased BNP levels. In contrast, intravenous Intralipid infusion, increasing the concentration of FFA, causes significantly increased BNP levels, abolishing the effect of hyperinsulinemia. Our results are consistent with a study also conducted using a euglycemic insulin clamp by Halbirk et al. [42]. It proved that a glucose-insulin-potassium infusion caused a decrease in NT-proBNP levels, possibly either by impaired synthesis or by decreased release from the myocardium. According to these results, insulin decreases NT-proBNP concentrations in healthy patients. It is worth mentioning that insulin has an opposite effect to BNP. Insulin is a hormone that keeps sodium in the body [43], while BNP is a natriuretic factor. It is also well known that insulin inhibits lipolysis in adipocytes in contrast to BNP, which is known to be potent stimulator of lipolysis in adipose tissue. However, intravenous insulin infusion in patients with type 2 diabetes does not change NT-proBNP concentrations [44], which may be due to insulin resistance. Furthermore, the experimental studies indicated that the gene expression of BNP is decreased in cardiomyocytes exposed to prolonged high doses of insulin [45]. As mentioned, increased FFA induces insulin resistance, which may also be related to an opposite effect on circulating BNP [46].

Our results show a negative correlation between OPG and BNP in the studied group, which is independent of BMI. Moreover, FFA increases the concentration of BNP and lowers the levels of OPG. There are many studies in the literature describing a positive correlation between OPG and NT-proBNP in patients with heart diseases [2,22,47,48]. It should be noted that with severe disorders, there is a significant increase in inflammation and an increase in glucose levels [49], which can affect the metabolism of OPG [50]. Thus, the presence of more severe metabolic disorders may lead to different results from our study with patients with normal glucose. To sum up, the negative correlation of BNP and OPG in young, healthy people means that these substances do not work synergistically, as suggested by the abovementioned authors. However, with the development of metabolic disorders, a compensatory increase in OPG may occur, leading to the development of a positive correlation between these substances. At present, the topic is relatively insufficiently understood and it requires further clinical and laboratory studies to better understand the relationship between BNP and OPG in patients without serious metabolic disorders.

## 5. Conclusions

Our data show that OPG and BNP are differentially regulated by FFA, which implies their association with lipid-induced insulin resistance. The proper assessment of these cardiovascular risk factors should take into account both long-term and short-term effects associated with insulin resistance.

## Figures and Tables

**Table 1:** Clinical and biochemical characteristics of the study groups.

	Normal-Weight (*n* = 30)	Overweight/Obese (*n* = 29)
Age (years)	23.20 ± 2.65	23.79 ± 2.55
BMI (kg/m^2^)	22.77 ± 1.58	28.72 ± 3.05 *
Waist circumference (cm)	82.38 ± 4.41	97.14 ± 10.27 *
% body fat	14.23 ± 3.93	25.67 ± 7.05 *
Systolic blood pressure (mmHg)	122.0 ± 7.72	127.96 ± 6.38 *
Diastolic blood pressure (mmHg)	77.1 ± 6.09	81.0 ± 6.9 *
Fasting plasma glucose (mg/dL)	84.21 ± 8.33	84.17 ± 7.63
Fasting serum insulin (μIU/mL)	8.51 ± 3.80	13.13 ± 4.80 *
Insulin sensitivity (mg/kg ffm/min)	7.36 ± 2.54	5.82 ± 1.92 *
Cholesterol (mg/dL)	163.23 ± 34.10	177.96 ± 30.76
Triglycerides (mg/dL)	76.67 ± 28.17	104.84 ± 58.90 *
HDL-cholesterol (mg/dL)	59.49 ± 8.22	54.41 ± 7.98 *
LDL-cholesterol (mg/dL)	99.06 ± 37.16	104.96 ± 28.49

* *p* < 0.05 vs. the normal-weight group.

## Data Availability

Not applicable.
